# Progressive Retinal Atrophy in the Border Collie: A new XLPRA

**DOI:** 10.1186/1746-6148-4-10

**Published:** 2008-03-03

**Authors:** Thierry Vilboux, Gilles Chaudieu, Patricia Jeannin, Delphine Delattre, Benoit Hedan, Catherine Bourgain, Guillaume Queney, Francis Galibert, Anne Thomas, Catherine André

**Affiliations:** 1 IGDR CNRS, Génétique et Développement, Faculté de Médecine, Université de Rennes1, 35043 Rennes Cedex, France; 2 Clinique vétérinaire, 2 Place Beaulieu F-63400 Chamalières, France; 3 INSERM U535, Génétique Epidémiologique et Structures des Populations Humaines, Hopital Paul Brousse, Villejuif, France; 4 Antagene, Immeuble Le Meltem, 2 Allée des Séquoias F-69760 Limonest, France

## Abstract

**Background:**

Several forms of progressive retinal atrophy (PRA) segregate in more than 100 breeds of dog with each PRA segregating in one or a few breeds. This breed specificity may be accounted for by founder effects and genetic drift, which have reduced the genetic heterogeneity of each breed, thereby facilitating the identification of causal mutations. We report here a new form of PRA segregating in the Border Collie breed. The clinical signs, including the loss of night vision and a progressive loss of day vision, resulting in complete blindness, occur at the age of three to four years and may be detected earlier through systematic ocular fundus examination and electroretinography (ERG).

**Results:**

Ophthalmic examinations performed on 487 dogs showed that affected dogs present a classical form of PRA. Of those, 274 have been sampled for DNA extraction and 87 could be connected through a large pedigree. Segregation analysis suggested an X-linked mode of transmission; therefore both XLPRA1 and XLPRA2 mutations were excluded through the genetic tests.

**Conclusion:**

Having excluded these mutations, we suggest that this PRA segregating in Border Collie is a new XLPRA (XLPRA3) and propose it as a potential model for the homologous human disease, X-Linked Retinitis Pigmentosa.

## Background

Progressive Retinal Atrophy (PRA) has been described in more than 100 breeds of dog [1-3], providing a powerful resource for the identification of new retinopathy-causing genes and a unique model for treatments for homologous human retinal diseases [4,5]. The strong founder effect and genetic drift occurring during the breeding of dogs may have significantly reduced the genetic heterogeneity of diseases in each breed, making it easier to identify causal mutations in dogs than in humans. Several genes responsible for canine retinopathies have yet been identified (Table 1). We focused on PRA, a clinically homogeneous group of diseases characterized by a loss of night vision in the first few years of life (2 to 5 years). This night blindness is followed by a progressive loss of the peripheral visual field and finally a total loss of vision, involving an initial loss of rods and then cone photoreceptors [2,6].

**Table 1:** Genes involved in canine retinopathies and in the Collie Eye anomaly, specifying the affected breeds and mutations.

**Gene**	**Chromosome**	**Disease**	**Affected breeds**	**Mutation**	**References**
*PDE6B* Phosphodiesterase *β *subunit	CFA3	Rod cone dysplasia Type 1 (rcd1 et rcd1a)	Irish Setter (rcd1)	Nonsense W807X	[37]
			Sloughi (rcd1a)	8 nucleotide insertion	[38]
*PDE6A* Phosphodiesterase *α *subunit	CFA4	Rod cone dysplasia Type 3 (rcd3)	Cardigan Welsh Corgi	Del A1939	[39]
*RHO* Rhodopsin	CFA20	Dominant PRA	English Mastiff Bullmastiff	T4R T4R	[40] [41]
*PDC* Phosducin	CFA7	Photoreceptors dysplasia (pd) Type A-PRA	Miniature Schnauzer	R82G	[42]
*RPGR* Retinitis Pigmentosa GTPase Regulator	CFA X	X-Linked Progressive Retinal Atrophy 1 (XLPRA1)	Samoyed Siberian Husky	Del GAGAA	[9]
		X-Linked Progressive Retinal Atrophy 2 (XLPRA2)	Mongrel	Del GA	[9]
*CNGB3* Cyclic Nucleotide Gated channel *β*3	CFA29	Achromatopsia-3	Alaskan Malamute	Deletion removing all exons (Del 140 kb)	[43]
			German Shorthaired Pointer	D262N	[43]
*RPE65* Retinal Pigment Epithelium-specific protein 65 kDa	CFA6	CSNB Congenital stationary night blindness	Briard	DelAAGA	[44]
*NHEJ1* NonHomologous End-Joining factor1	CFA37	CEA Collie Eye Anomaly	8 breeds (1)	7.8 kb deletion	[31]
*PRCD* Progressive Rod Cone Degeneration	CFA9	PRCD Progressive Rod-Cone Degeneration	22 breeds (2)	C2Y	[7]
*RPGRIP1* RPGR Interacting Protein 1	CFA15	CORD1 Cone Rod Dystrophy 1	Miniature Longhaired Dachshund	44 nucleotide insertion	[45]
*BEST1* Bestrophin 1	CFA18	CMR Canine Multi-focal Retinopathy	5 breeds (3)	R25X G161D	[46]

(1) Australian Shepherd, Border Collie, Lancashire Heeler, Nova Scotia DTR, Rough Collie, Shetland Sheepdog, Smooth Collie, Whippet: longhaired.

(2) American Cocker Spaniel, American Eskimo Dog, Australian Cattle Dog, Australian Shepherd, Australian Stumpy Tail Cattle Dog, Chesapeake Bay Retriever, Chinese Crested, Cockapoos, English Cocker Spaniel, Entelbucher Mountain Dog, Finnish Lapphund, Golden Retriever, Kuvasz, Labradoodle, Labrador Retriever, Lapponian Herder, Nova Scotia Duck Tolling Retriever, Poodle Miniature and Toy, Portuguese Water Dog, Spanish Water Dog, Swedish Lapphund.

(3) English Mastiff, Bullmastiff, Dogue de Bordeaux, Great Pyrenees, Coton de Tulear

Age-at-onset differs between breeds. PRA are also highly heterogeneous genetically, with several modes of transmission and a large number of genes and mutations involved. Each PRA generally occurs in only one or a few breeds, as demonstrated for PRA with a known genetic basis [3] (Table 1). PRA-prcd is a notable exception, affecting more than 20 breeds [7,8]. Only two X-linked PRA have been described both involving the *RPGR *gene (Retinitis Pigmentosa GTPase Regulator) with a different mutation in exon 15 (ORF15) in each breed. XLPRA1 is caused by a deletion of five nucleotides, leading to a frameshift and immediate premature stop in the Siberian Husky and Samoyed. XLPRA2 is caused by a deletion of two nucleotides leading to a frameshift that has been shown to result in significant changes in the deduced peptide sequence in a mongrel dog [9].

PRA are naturally occurring retinal diseases in dogs, and have a phenotype similar to that of Retinitis Pigmentosa in humans. Retinitis Pigmentosa (RP) is the most prevalent group of inherited retinopathies in humans, affecting about 1 in 3600 individuals [10]. RP display considerable clinical and genetical heterogeneity, with wide variations in disease onset, progression and severity [11] and several transmission modes. Up to now, 54 loci for non-syndromic RP have been mapped, for which 39 genes have been identified [12]. Those genes account for an estimated 50% of dominant RP, 40% of recessive RP and 80% of X-linked RP cases [13].

Studies aiming to identify the genes responsible for X-linked RP have led to the identification of four loci (RP6, RP23, RP24, RP34) and only two genes (*RPGR *and *RP2*) [14-19]. Other genes for X-linked RP remain to be identified, indeed, known genes and loci involved in those diseases have been excluded in several families [20]. The *RPGR *gene, a GTPase regulator that is essential for the maintenance of photoreceptor viability, is involved in the X-linked RP3 disease. In this gene nearly 100 mutations have been already described in several families [21]. The *RP2 *gene responsible for X-Linked RP2 disease is thought to be involved in the beta-tubulin folding [17]. Up to 17 mutations have been identified as associated with RP2 [21]. In the last decade, the canine model has displayed considerable genetic potential, as individual breeds correspond to isolated populations, it has facilitated the identification of a number of dog genes and invaluable candidates for the homologous diseases in humans [22,23]. This applied to retinal diseases and PRA in particular, with the example of the identification of a new canine gene (*PRCD*), responsible for PRA-prcd in different breeds. This gene, which was not annotated in the human genome, constituted a new candidate gene for human RP and indeed, a mutation in this gene has been identified in a patient from Bangladesh with RP [7].

We searched for potential candidate genes for human RP, by investigating a PRA segregating with a high frequency in the Border Collie breed. Three retinopathies have been described in Border Collie: the Colley Eye Anomaly (CEA) [24], the Central Progressive Retinal Atrophy (CPRA) [25,26] and the (non central) Progressive Retinal Atrophy (PRA) [27]. Border Collie belongs to the Collie lineage in which CEA is frequently diagnosed in Colley but less frequently in Border Collie. The primary clinical sign in CEA is choroidal hypoplasia corresponding to the under-development of the choroid. Moderately affected dogs present a normal vision throughout life but in severely affected individuals, colobomas at the optic nerve can lead to retinal detachments and blindness [24]. CPRA has an incidence of 12% in the breed in the United Kingdom, its occurrence is between 1 and 2 years of age, with primary brown spots in the tapetal area along the vessels, the later stages looking like PRA. The both eyes were generally identically affected and secondary cataract is rarer than in PRA [25,26]. The PRA that occurs in the Border Collie has been clinically described previously [27]. In the present study, the clinical examination of 487 Border Collie dogs from France demonstrated the occurrence of PRA at a high frequency in this breed. We present a precise clinical description of this canine PRA and the first steps of the genetic analysis. The genetic study consisted in collecting cases and controls as well as related individuals to investigate the exact mode of transmission and to test known canine mutations. Over 250 Border Collie blood samples were collected, most of them could be connected through a 375 dog pedigree. Segregation analysis suggested an X-linked mode of transmission. We investigated the possibility of the molecular cause of this PRA being a known mutation, by carrying out the genetic tests for XLPRA1 and XLPRA2. The exclusion of these mutations as possible causes of the disease suggested that this PRA in Border Collie might correspond to a new form of X-linked PRA. We are currently collecting a larger sample of dogs for linkage analysis.

## Results

### Clinical features of PRA in the Border Collie

In total, 487 dogs were examined by the same veterinary ophthalmologist. All of them are working Border Collies and were examined all over France, for purpose of clinical prediction of inherited eye diseases. In total, the 241 males and 246 females were aged of 2 months to 13 years, 293 of which were at least 2 years old at the time of the first examination (mean age at the first examination: 3.25 years); 357 dogs were examined once, 86 twice, 23 three times, 17 four times, 4 five times, on average in the space of a one year period.

Clinical examination consisted in the evaluation of menace responses and pupillary light reflexes and indirect ophthalmoscopic examination (30*δ *& 20*δ *lenses) after pupillary dilatation. A total of 60 dogs (54 males, 6 females) aged between six months and nine years (mean age of diagnostic: 3.85 year old) presented lesions of the fundus, mostly in the tapetum: 31 dogs were bilaterally affected, 26 dogs were unilaterally affected at the first examination and bilaterally at the second examination and three dogs were unilaterally affected at the second examination and bilaterally at the third examination.

**Figure 1:**
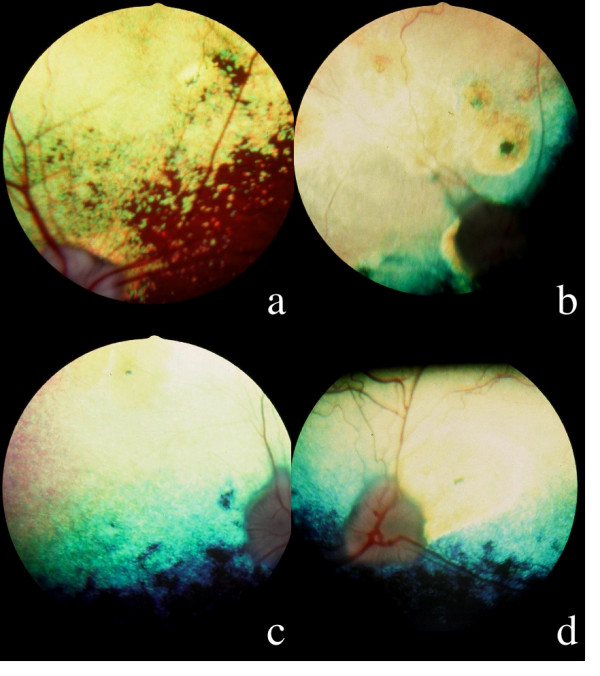
**Ophthalmoscopy; 1a: hyperreflective nasal tapetal focal lesion with a pigmented dot in a 3 years old affected male, OD: the optic disc, the vessels and the nontapetal area are still remaining normal.** 1b: Multifocal coalescent hyperreflective lesions in a 3,5 years old affected male: a pigmented dot is visible at the center of a suprapapillar lesion; the tapetum appears hyperreflective due to the coalescence of multiple focal lesions; the optic disc looks grayish and the arterial vasculature has disappeared. 1c: Generalized hyperreflexion of the tapetum in a 3 years old affected male, OD: the tapetum appears hyperreflective, with the exception of a subnormal horizontal band close to the nontapetal area; the optic disc is grayish and the arteries have disappeared. 1d: OS, dog of the figure 1c: attenuation of the arterial diameter, temporal triangular suprapapillar hyperreflective area with a pigmented line at its center.

Lesions of the fundus were variable: at early stages, focal or multifocal hyperreflective lesions of the tapetum resulting in thinning of the retina, with an inconstant symmetry were frequently observed (Figure 1a), but the hyperreflective band surrounding the tapetal-nontapetal junction was rare; at advanced stages, generalized hyperreflexion coexisted either with hyperreflective coalescent foci in the tapetum (Figure 1b), or with diffuse tapetal hyperreflexion (Figure 1c) and depigmentation/hyperpigmentation in the nontapetum (Figure 1c and 1d), whereas the vessels became thinner before disappearing and the optic disc became grayish (atrophic myelin loss) (Figure 1b and 1c); secondary cortical equatorial cataracts appeared after the age of seven years, but this finding was rare in the affected dogs, some of which were totally blind [28].

The correspondence between ophthalmoscopic examinations and evaluation of menace responses and pupillary light reflexes was the following:

#### Observations for unilateral lesions

Isolated peripheral or central hyperreflective or grayish focal tapetal lesions, with or without a pigmented dot at their centre: pupillary light reflexes and menace response were present in all dogs.

Multiple hyperreflective focal lesions with diffuse increased in tapetal reflexion, thinning of the vessels and multifocal depigmentation of the non tapetal zone: the menace response was present but pupillary light reflexes were delayed and incomplete.

#### Observations for bilateral lesions

Isolated symmetric focal hyperreflective tapetal lesions, with a pigmented center and focal discolored non tapetal lesions: the menace response was present but pupillary light reflexes were delayed and incomplete;

Symmetric horizontal hyperreflective tapetal band parallel to the tapetum/non tapetum juction: the menace response and pupillary light reflexes were present and unmodified;

Multiple symmetric hypereflective tapetal lesions and multifocal discolored non tapetal lesions: at reevaluation one year later, these dogs displayed either additional similar lesions or generalized atrophy of the fundus (coalescent lesions) with hyporeflective midriasis and thinning of the vessels in all cases and palor of the papilla in some cases; all these dogs had lost the menace response.

#### Electroretinography results

**Figure 2:**
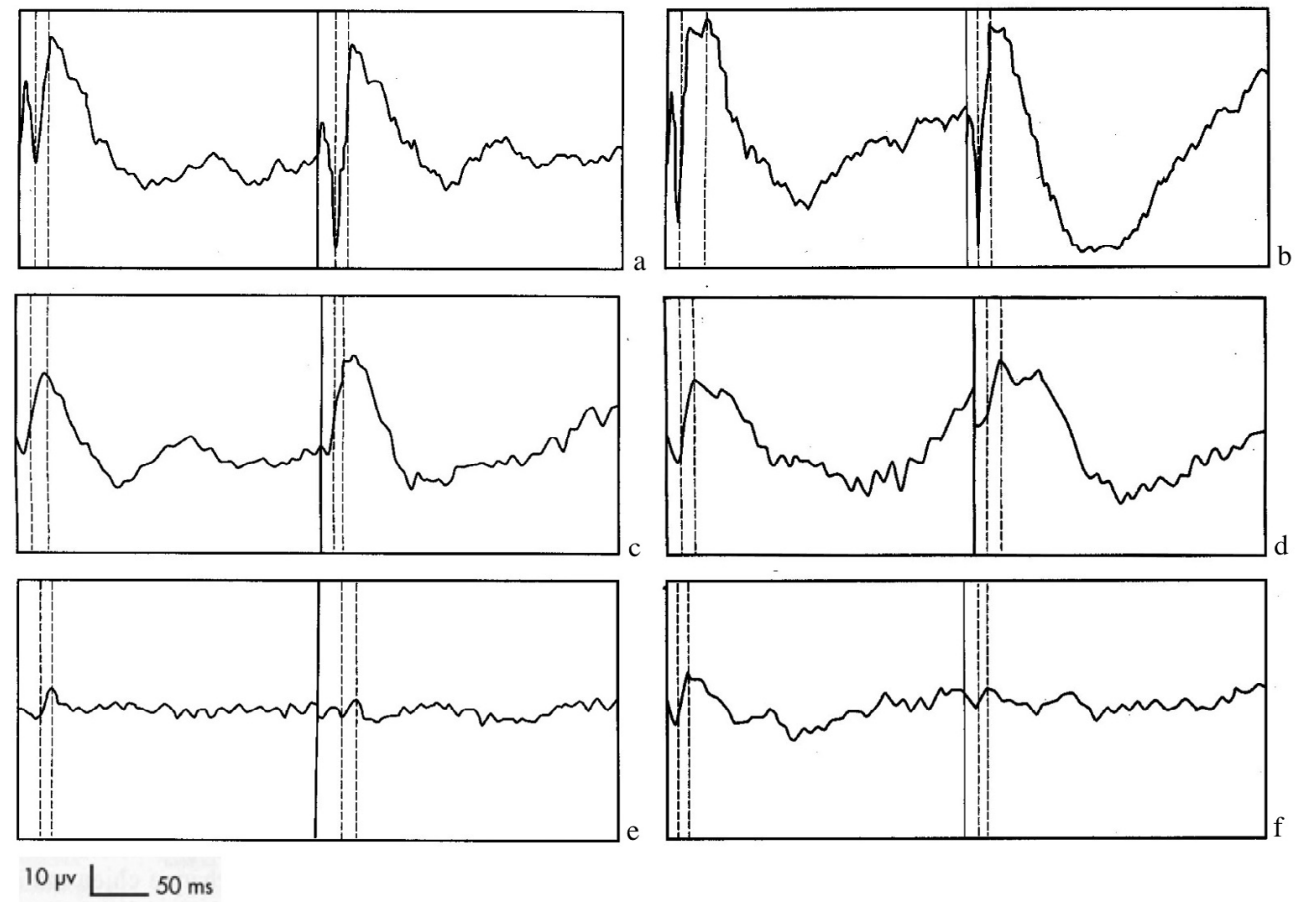
**Electroretinograms; 2a, 2c and 2e: dark adaptation, blue light scotopic stimuli.** 2b, 2d and 2f: dark adaptation, white light stimuli. 2a and 2b: 2 years old unaffected male. 2c and 2d: 3 years old affected male with isolated lesions. 2e and 2f: 9 years old affected dog with generalized lesions.

Electroretinography (ERG) was carried out as a complementary examination in 15 dogs (14 affected, 1 clinically unaffected but at risk). Based on recordings in the same conditions for a two-year-old male dog used as a reference, we found: normal traces in cases of isolated unilateral peripheral or central hyperreflective or grayish lesions; significant to severe decrease in a and b-wave amplitude and in some cases, non recordable scotopic blue ERGs when symmetric multifocal hyperreflective tapetal lesions or diffuse hyperreflective tapetum were observed (Figure 2). A severe decrease in b-wave amplitude in scotopic blue and achromatic white stimulations; an increase in culmination phases when multiple symmetric hyperreflective focal lesions or diffuse hyperreflective tapetal zones were present.

#### Fluorescein Angiography results

Fluorescein angiography (FA) was performed in 10 affected dogs. Dogs were declared unaffected if they remained asymptomatic after the age of five years. Younger dogs without symptoms were considered to have an unknown phenotype.

**Figure 3:**
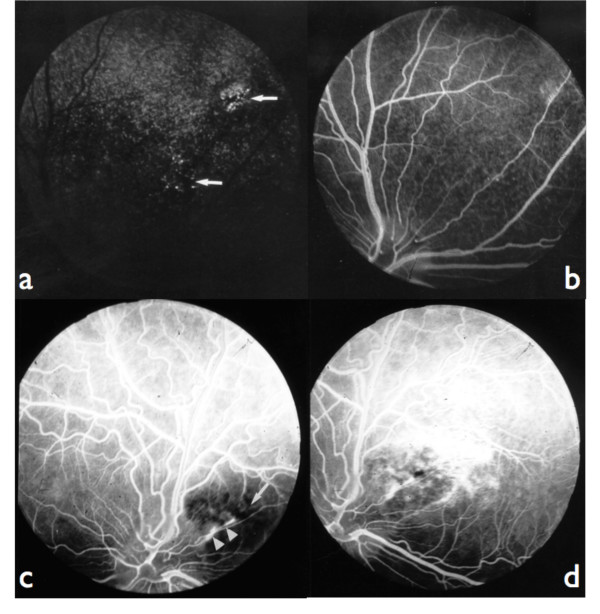
**Fluorescein angiograms: 3a: fundus of the figure 1a, choroidal phase: combined window effect and masking effect (arrows).** 3b: fundus of the figure 1a, late venous phase: epithelial fluorescein leakage at the level of lesions. 3c: fundus of the figure 1d, laminar venous phase: masking effect by retinal pigment at the level of the lesion (arrow); linear leakage of fluorescein in the pigment epithelium (points of arrows). 3d: fundus of the figure 1d, late venous phase: most important diffusion of fluorescein at the periphery of the lesion; the hyperfluorescent lines (linear leakage) are corresponding to localized ruptures of the Bruch's membrane.

**Figure 4:**
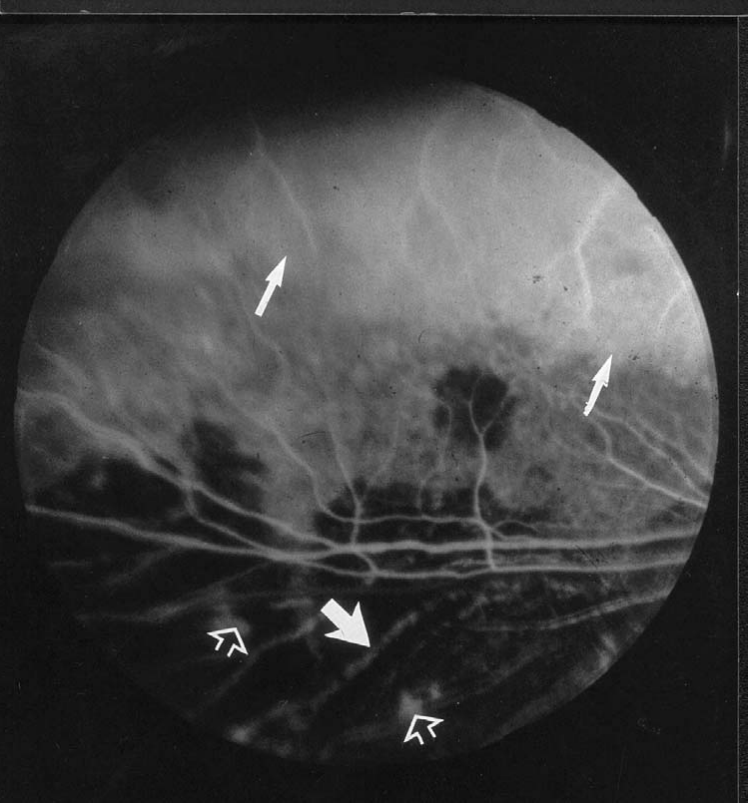
Fluorescein angiogram; 7 years old affected male, late venous phase: epithelial leakage of fluorescein in the tapetal area (arrows) and in the nontapetal area (hollowed arrow), visible choroidal vasculature (bold arrows).

We observed either hypofluorescent or hyperfluorescent defects as follows:

early focal hyperfluorescence in the tapetum by window defect, which remained without any modification or became wider through peripheral epithelial fluorescein leakage throughout the angiogram (Figure 3a and 3b);

constant focal hypofluorescence linked to masking effect by retinal pigment at the level of the lesion and progressive diffusion of the fluorescein by leakage in the pigment epithelium (Figure 3c);

linear ruptures of Bruch's membrane in the area centralis (Figures 3c and 3d);

epithelial fluorescein leakage starting from neovessels either in the tapetum or nontapetum and increasing during late phases of the angiogram in advanced atrophic retinal lesions (Figure 4).

All these lesions corresponded to primary retinal involvement, but were not suggestive of CPRA, neither of XLPRA 1. It does not either correspond to XLPRA2 since, in XLPRA2, retinal development is aberrant with ERG abnormalities at as early as 5–6 weeks of age. At the age of 4 months retinal degeneration rapidly progresses and by 2 years, retina is fully degenerated in all affected dogs [9] whereas in the Border Collie PRA, the clinical signs are at their beginning when observed.

### Pedigree

**Figure 5:**
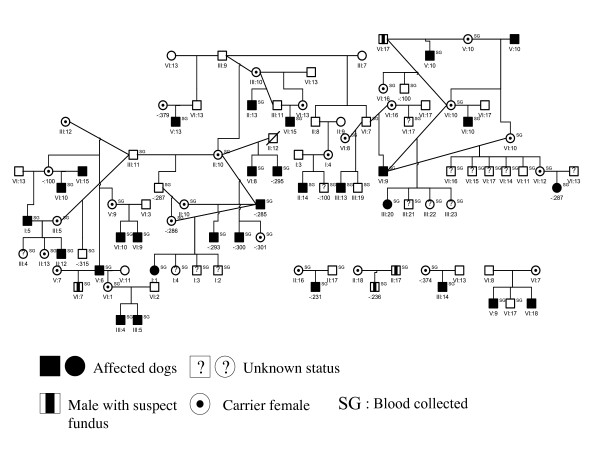
Illustration of a Border Collie pedigree segregating PRA constructed by the Cyrillic 2.1 software. This pedigree is constituted of 80 dogs, 33 dogs are affected (30 males and 3 females).

Out of the 487 clinically examined dogs, a subset of 80 Border Collies (33 affected, 30 males and 3 females) belonging to a large pedigree were sampled for the genetic analysis. By retrieving the genealogical data of these 80 dogs, a pedigree of 375 individuals was constituted using the Cyrillic 2.1 software but no common ancestor could yet be defined (Figure 5). As previously stated, all dogs are working Border Collies sampled from all over France and no bias is suspected in the sampling.

### Segregation analysis

For inference of the genetic model best accounting for the observed segregation of the disease in the 375-dog pedigree, we performed a segregation analysis using the Generalized Linear Model approach implemented in Mendel 6.1. We chose this relatively simple segregation approach because a highly penetrant X-linked model was suspected in the family and because the use of Mendel6.1 made it possible to analyze the pedigree in all its complexity. The results are presented in Table 2. We first compared the likelihood of the general autosomal model with that of the X-linked model using the AIC criterion, as these two models are not nested. The general X-linked model fitted the data better (AIC_X-link _= 88.4 vs AIC_autos _= 110.6). If we constrained the X-linked model to either a general dominant (f12 = f22) or a general recessive model (f11 = f12), the parameter estimates converged to give a fully penetrant recessive model with a risk allele frequency = 0.46. The chi2 statistic for comparison of the fully penetrant X-linked recessive model with the general X-linked model was not statistically significant (pval = 0.10). Consequently, the fully penetrant X-linked recessive model with a risk allele frequency of 0.46 is the most parsimonious model best fitting the data.

**Table 2:** Segregation analysis – Parameter estimates, log-likelihood and AIC associated with the general autosomal mode of transmission and the general, dominant and recessive X-linked modes of transmission.

	**Autosomal**	**X-linked**		
	**General**	**General**	**Dominant**	**Recessive**

**q**_2_	0.39	0.39	0.54	0.46
***f***_11_	0.38	0.29	1	0
***f***_12_	0.31	0	0	= *f*_11_
***f***_22_	0.41	0.88	= *f*_12_	1
**No.**	4	3	1	1
**-2 lnL**	102.6	82.4	87	87
**AIC**	110.6	88.4	89	89
***χ***^2^**(df)**			4.6(2)	4.6(2)
***P***			0.10	0.10

q_2_: frequency of the risk allele 2; *f*_11_, *f*_12_, *f*_22_: penetrances of genotypes 11, 12 and 22. No.: No. of independent parameters estimated = (no. of parameters in the model)-(no. of parameters that converged to a boundary); lnL: log-likelihood; AIC: Aikaike criterion (-2 lnL + 2 No). *χ*^2 ^(df): chi-squared test comparing the general X-linked model with the restricted X-linked models. *P*: associated p-value.

### Mutation screening

#### XLPRA1 and XLPRA2

In order to check if the PRA in the Border Collie could be due to the XLPRA1 or XLPRA2 mutations of the *RPGR *gene, we tested 36 PRA affected Border Collie for XLPRA1 and 34 affected Border Collie for XLPRA2. None of those mutations were detected in any dog.

#### CEA mutation

The Border Collie is one of about a dozen breeds of the Collie lineage [29], susceptible to be affected by the Collie Eye Anomaly (CEA) disease [30]. The frequency of this disease depends on the breeds and the countries [24], being rarely detected in France, clinical signs of CEA were found in only one dog of 1000 examined (Chaudieu, personal communication). Clinical investigations were not suggestive of CEA in any of the dogs, but 24 dogs, including 20 affected dogs, were nonetheless screened for the CEA mutation in the *NHEJ1 *gene [31] to exclude the possible co-segregation of CEA and PRA at the molecular level. This mutation was not detected in any of the 24 dogs tested.

#### PRA-prcd

The PRA-prcd mutation segregates in a large number of breeds, indeed, it has been found in 22 breeds [7]. To exclude the co-segregation of this mutation in Border Collies, we tested 55 dogs including 34 affected dogs for the prcd mutation in the *PRCD *gene [32]. This mutation was not detected in any of the 55 dogs tested.

As no known mutations for retinopathies likely to affect Border Collies were found in any of the dogs tested, we suggest that the PRA segregating in this breed corresponds to a new XLPRA (XLPRA3).

## Discussion

In the present study, we described a new canine retinopathy. The clinical form described here is clearly characteristic of a progressive retinal atrophy (PRA): the onset of symptoms occurred at around three years of age, and peripheral vision was then progressively lost over the next years. The appearance of the fundus was compatible with PRA with distinct peripheral pigmentary changes, optic pallor, and attenuated retinal vessels. ERG responses were suggestive of the disease in two-year-old dogs. The predominant symptoms of night blindness, peripheral field defects, and absence of light sensitivity strongly suggest that this is a form of PRA [25,26].

All the clinical signs that we observed (menace responses, pupillary light reflexes, fundus examination) were evocating a PRA:

- focal lesions were evoluting from one eye to both in cases of unilateral lesions at the first examination;

- either focal or diffuse bilateral lesions were evolving towards a generalized disease of he fundus in re-examined dogs, with PRA compatible signs: tapetal hyperreflexion, vascular attenuation, palor and enlargement of the optic disc;

- vacuolar subcapsular equatorial cortical cataracts observed in some dogs with severe fundic lesions were described in other canine breeds as secondary to PRA [6,27];

- the most important was midriasis, the most incomplete, unstable and the slower was the pupillary light reflex, with disappearing of the menace response in cases with severe fundic lesions.

If only focal isolated lesions were observed, there was no modification of the ERG. In cases of multiple or generalized fundic lesions, the hypovolted traces (sometimes non recordable in blue stimulation) were in favour of a progressive rod and cone degeneration.

We never found either ophthalmoscopic, functional or angiographic signs which correlated with CPRA: this disease was first described in several breeds, considered as recessive autosomal in the Briard shepherd dog, dominant autosomal with incomplete penetrance in the Labrador Retriever, the Shetland sheepdog [25-27], dominant autosomal with incomplete penetrance [26] or polygenic [25] in the Border Collie. Second, it appears that environmental factors were involved in CPRA: experimental Vitamin E deficiency exactly mimicked the same fundic lesions in the dog [33]. Nevertheless, in Border Collies, the results of eye examination scheme in the United Kingdom remained in favor of an hereditary origin: in 1965, 12% of the dogs were affected of CPRA; in 1989, only 2% were exhibiting ophthalmoscopic changes characteristic of the disease [25,26]. All the findings of FA were demonstrating that the primary changes in the fundus were not related to a pigment epithelium dystrophy: we never found the specific lesions of fluorescence mottled masking by lipofuscinic like pigment deposits [34]. We showed that this PRA segregates according to an X-linked mode of transmission. This is compatible with the status of the few affected females identified. Indeed, the six females found affected, after clinical examination, were diagnosed between 18 months and 3 years old and clinical symptoms were very similar to those observed in males. Moreover, all females presented similar clinical features. For three of them, we do not have any genealogical data and thus we do not know the status of the parents. For the other three affected females, which are in the pedigree, they were all born from an affected father and an obligate carrier female. Considering the high level of inbreeding in the breed, we assume that affected females come from obligate carriers and affected males, supporting a fully penetrant recessive inheritance mode. Genetic tests carried out on subsets of dogs excluded the involvement of known mutations.

We excluded the XLPRA1 and XLPRA2 mutations as the possible causal mutation for this X-linked PRA. However, owing to differences in the clinical signs and age-of-onset observed with XLPRA2, the second ORF15 mutation in *RPGR *could likely be excluded [9]. The CEA mutation is frequent in the Collie lineage, but occurs at only low frequency in the Border Collie breed in France (Chaudieu, personal communication). We nonetheless tested for the presence of the CEA mutation that could co-segregate. As expected, the dogs analyzed did not carry the mutation, consistent with the low prevalence of CEA in the Border Collie. Finally, we also tested for the presence of the mutation for *PRCD*, which has been involved in many dog breeds displaying PRA-prcd, while these breeds have no evident common origin [7]. Neither CEA, nor prcd were found to co-segregate in affected Border Collies.

In human, retinitis pigmentosa occurs and presents clinical features similar to canine PRA. Human cases experience a decrease in peripheral and night vision in their twenties, then night blindness and diminution of the visual field in their thirties, but visual acuity and color vision may remain normal until advanced stages of the disease [13]. Clinically, X-linked RP are the most severe forms of RP [13] but only 2 genes have yet been identified on the X chromosome. Human families are often too small for the identification of the causal loci by linkage studies and families cannot be grouped for genetic linkage analyses because of possible genetic heterogeneity. Indeed, several causal genes have already been identified in inbred families [12]. As dog pedigrees are highly inbred, it may be a useful model to identify new candidate genes for X linked RP.

The pedigree used for the segregation analysis is currently being completed for genetic linkage analysis to search for the genetic locus involved in the disease. The XLPRA1 and XLPRA2 mutations are not involved in this XLPRA, but we cannot exclude the involvement of other mutation in the *RPGR *gene. Indeed, this is the only gene involved in canine XLPRA with two mutations presently described in exon 15. In human, *RPGR *is frequently and differently mutated in XLRP. Other human candidates, such as the XLRP gene (*RP2*) or loci (RP6, 23, 24, 34) [14-19] may be analysed. We expect linkage analysis to be more efficient to test the involvement of genes and loci, as gene sequencing cannot unequivocally exclude the involvement of a gene if no mutation is found especially as some regions are usually unscreened, such as introns, 5' and 3' UTR or other regulatory regions.

## Conclusion

Studies of this new XLPRA should lead to the identification of a new mutation in one of the previously identified genes or in a new gene on the X chromosome in dog. This disease could constitute a model for X-linked human RP. As 20% of XLRP genes remain to be identified in human [13], the Border Collie provides an opportunity to identify a new causal gene thus representing a potentially pertinent model for studying the genetic bases of XLRP in human.

## Methods

All experiments have been performed under agreement reference number A35-238-13 delivered in July 2003 by the animal healthcare and protection service of the French government.

### Clinical investigation/ophthalmologic examination

None of the dogs was kept for research purposes. All were privately owned sheep dogs.

The 487 dogs were examined both by evaluating pupillary light reflexes and menace response and by indirect ophthalmoscopy (30*δ *& 20*δ *lenses) after pupillary dilation (0.5% Tropicamide drops, Mydriaticum^®^, Théa, Clermont-Ferrand Cedex, France).

#### Electroretinography (ERG)

ERG included the following procedures: pupillary dilatation (Mydriaticum^®^); dark adaptation for 15 min; general anesthetic (2% xylazine, Rompun^®^, Bayer Pharma, Puteaux cedex – France: 0,05 mL/kg and 10% ketamine chlorhydrate, Imalgene 1000^®^, Merial, Lyon – France: 0,05 mL/kg) administered IV; 10 achromatic stimulations for each eye with a white standard flash (2,5 cd/s/m^2^), at a 1 Hz frequency; 10 chromatic stimulations for each eye with a blue filter (440 nm) at a scotopic level and a 1 Hz frequency; signals recorded with needle electrodes (active and reference electrodes for each eye).

#### Fluorescein Angiography (FA)

FA was performed without sedation after pupillary dilatation (Mydriaticum^®^) using a Kowa – RC fundus camera (Kowa Company, Tokyo, Japan) fitted with a specially adapted blue stimulation blue filter, yellow filters and loaded with Ilford HP5 ISO 400 black-and-white film (Ilford Imaging, Mobberley, UK); 10% Sodium fluoresceinate (2 mL/10 kg bodyweight, Ciba Vision Ophthalmics, Toulouse – France) was injected intravenously, when starting the examination of the fundus.

### Pedigree analysis

A 375-dog multigeneration pedigree, connecting 87 dogs with known phenotypes was constructed. 33 dogs are affected (30 males and 3 females). This pedigree is highly inbred with frequent marriage loops. We used the Cyrillic software v2.1 [35] (^© ^CyrillicSoftware) for genealogic and genetic data management.

All dogs subjected to genetic analysis for which parental DNA was available, were tested for parental compatibility using the ISAG reference markers.

### Genomic DNA extraction

Blood samples and the accompanying pedigree were collected by DVM veterinaries or based on information obtained directly from the owners. All data were entered into a database. Genomic DNA was extracted from 5 ml of blood collected into EDTA, using the nucleon BACC 3 kit (GE Healthcare Bio-Sciences Corp, Piscataway, NJ, USA). The extracted DNA was "Whole Genome Amplified" using the V1 Genomiphi kit (GE Healthcare).

### Segregation analysis

Segregation analysis was performed on a 375-dog pedigree, that corresponds partially to the diagnosed dogs, using the generalized linear model-based approach implemented in Mendel 6.1 [36]. We analyzed the qualitative phenotype under study, by considering the logit function as a link function in the model. Briefly, let p be the probability of being affected, conditional on the genotype and let X_11_, X_12_, X_22 _be binary variables describing the genotype at a biallelic disease locus (X_11 _= 1 and X_12 _= X_22 _= 0 for genotype 11). The general model is $\log\frac{\pi }{1-\pi }=\alpha +\beta_{1}X_{11}+\beta_{2}X_{12}+\beta_{3}X_{22}$. The different segregation models to be compared in the analysis are expressed as constraints on (*β*_1_, *β*_2_, *β*_3_). Likelihoods of the models are compared with likelihood ratio statistics when models are nested and using the AIC criterion (best model minimizes (-2 lnL + 2 No), where L is the Likelihood of the model and No the number of independent parameters estimated in the model) for non nested models.

### Mutation testing

Dogs of the pedigree were screened for the known mutations, XLPRA1 [9], prcd [7,32] and the mutation for Colley Eye Anomaly (CEA) [31] as described. For the XLPRA2 mutation, primers were designed using the *RPGR *ORF15 sequence (AF385629) [9]. The PCR were performed with DNA amplified by Genomiphi V2 (GE Healthcare).

Primers:

For XLPRA1 mutation [9]:

RGF14: AAGGGGAGGAGAAAGGGGAGGCT

RGR13: TCCCTCTTCCTCCTCCCCTTCATA

For XLPRA2 mutation:

XLPRA2U: AGAGGCAGAATGGGAAGGAA

XLPRA2L: CCGTCTTCCCTGTTTTTCAC

For CEA mutation [31]:

aF-D GGAGGAGTCATCATGACTTGC

aR-D GACTGGTATTATCAAAGGTCAC

For PRA-prcd mutation [32]:

prcd1f: CCAGTGGCAGCAGGAACC

prcd2r: CCGACCTGCTGCCCACGACTG

## Authors' contributions

TV collected samples, constructed the pedigrees and performed molecular analyses; he interpreted data and actively participated to the manuscript redaction. GC did all clinical diagnoses, collected samples and actively participated to the writing of the manuscript. PJ and CB performed the statistical analyses and interpreted the data; they critically revised the manuscript. DD performed genetic tests. BH extracted DNA from blood samples. GQ and FG gave critical advices on the work and manuscript. AT contributed to the conception and design of the work and to the writing of the manuscript. CA contributed to the conception and design of the work and drafted the manuscript.
